# Dr. Jacob Chambers

**Published:** 1904-10

**Authors:** 


Dr. Jacob Chambers, of Kingston, N. Y., a graduate of the University of Buffalo, 1875, died at his home September 16, 1904, aged 52 years. He was one of the managers and surgeons of the Kingston City Hospital and physician to the family of Judge Alton B. Parker, democratic candidate for the Presidency.

